# Supplementary material for: Quantitative Image Processing for Three-Dimensional Episcopic Images of Biological Structures: Current State and Future Directions
Source: Biomedicines. 2023 Mar 15;11(3):909. doi: 10.3390/biomedicines11030909 (PMC10045950; doi:10.3390/biomedicines11030909)
Supplement: Supplementary file 1 [file biomedicines-11-00909-s001.zip › biomedicines-2208275-supplementary.pdf]

Supplementary Materials

Table S1. Summary of papers included in meta-analysis.

Paper title	Year	Contrast	Structure	Model	Annotation type	Method of annotation	Type of quantification	Details of quantification	Software	Online image repository
Comparison of ex-vivo high-resolution episcopic microscopy with in-vivo four-dimensional high-resolution transvaginal sonography of the first-trimester fetal heart [31]	2011	Eosin	heart	human	Object detection	Manual	2	Counts of identifiable structures	Unknown	www.embryoimaging.org
A chick embryo with a yet unclassified type of cephalothoracopagus malformation, a hypothesis for explaining its genesis [32]	2012	Eosin	embryo	chick	Segmentation, Detection	Semi-automatic skeletonization, Manual	5	Length measurements	Amira	N/A
Congenital heart disease, the specification of left-right asymmetry [33]	2012	EFIC	heart	mouse	Object detection	Manual	2	Phenotype counting	Osirix	N/A
Dimensions of the Great Intrathoracic Arteries of Early Mouse Fetuses of the C57BL/6 Strain [34]	2012	Eosin	heart	mouse	Segmentation	Manual	4	Diameter measurements at defined locations	Amira	N/A
High-Resolution Episcopic Microscopy Data-Based Measurements of the Arteries of Mouse Embryos: Evaluation of Significance and Reproducibility under Routine Conditions [35]	2012	Eosin	heart	mouse	Segmentation	Manual, semi-automated protocol	4	Measurements at defined locations, virtual resectioning	Amira	N/A
IFT25 Links the Signal-Dependent Movement of Hedgehog Components to Intraflagellar Transport [36]	2012	EFIC	heart	mouse	Visualisation	N/A	N/A		Volocity	N/A
Lineage tree for the venous pole of the heart: clonal analysis clarifies controversial genealogy based on genetic tracing [37]	2012	Eosin + X-gal	heart	mouse	Object detection	Manual	2	Count of recombination event via X-gal labelling	Osirix	N/A
Model-based vasculature extraction from optical fluorescence cryomicrotome images [25]	2012	Cryo	heart	pig	Segmentation	Frangi filtering, Intensity thresholding	5	Skeletonisation of vessels	C, MATLAB, ImageJ, Fiji	N/A
Modifying transcript lengths of cycling mouse segmentation genes [38]	2012	Eosin	skeleton	mouse	Object detection	Manual	1	Somite count	ImageJ, Osirix	N/A
Multiscale analysis of coronary branching, collateral connectivity: coupling vascular structure and perfusion in 3D [26]	2012	Fluorescence	heart	human, pig	Segmentation	Frangi filtering	5	Deconvolution, Franji filtering	CUDA	N/A

Normal, abnormal development of the intrapericardial arterial trunks in humans, mice [39]	2012	Eosin	heart	mouse	Segmentation	Manual	N/A		Amira	N/A
Three-dimensional, molecular analysis of the arterial pole of the developing human heart [40]	2012	Eosin	heart	Human, mouse	Object detection	Manual	N/A		Osirix, photosho p	N/A
A new definition of left ventricular compaction/noncompaction-the new gold-standard? [41]	2013	Eosin	heart	mouse	Segmentation	Intensity based	6	Edge detection, fractal analysis	Unknown	N/A
ARMC4 mutations cause primary ciliary dyskinesia with randomization of left/right body asymmetry [42]	2013	EFIC	heart	mouse	Object detection	N/A	N/A		Osirix	N/A
Interrogating congenital heart defects with noninvasive fetal echocardiography in a mouse forward genetic screen [43]	2013	EFIC	heart	mouse	Object detection	Other	1	Phenotype counting	Unknown	N/A
Metric characterization of the aortic arch of early mouse fetuses and of a fetus featuring a double lumen aortic arch malformation [44]	2013	Eosin	heart	mouse	Segmentation	Manual, semi-automated protocol [45]	4	Diameter and area measurements	Amira	N/A
Microcomputed Tomography Provides High Accuracy Congenital Heart Disease Diagnosis in Neonatal and Fetal Mice [46]	2013	EFIC	heart	mouse	Object detection	Manual	2	Phenotype counting	Unknown	N/A
The dermal arteries of the human thumb pad [10]	2013	Eosin	dermal arteries	human	Segmentation	Manual	N/A	Manual vessel tracing	Amira	N/A
Ultra-high frequency ultrasound biomicroscopy and high throughput cardiovascular phenotyping in a large scale mouse mutagenesis screen [47]	2013	EFIC	heart	mouse	Visualisation	Manual	N/A		Unknown	N/A
Wdpcp, a PCP protein required for ciliogenesis, regulates directional cell migration, cell polarity by direct modulation of the actin cytoskeleton [48]	2013	EFIC	heart	mouse	Object detection	N/A	N/A		Unknown	N/A
A detailed comparison of mouse and human cardiac development [49]	2014	EFIC	heart	mouse	Object detection	N/A	N/A		Volocity, OpenLab	http://apps.devbio.pitt.edu/MouseAtlas, http://apps.devbio.pitt.edu/HumanAtlas.
Advanced assessment of cardiac morphology and prediction of gene	2014	Eosin	heart	mouse	Segmentation	Fractal analysis	6	Fractal analysis (box counting)	Unknown	N/A

carriage by CMR in hypertrophic cardiomyopathy-the HCMNet/UCL collaboration [50]										
Characterizaton of the vessel geometry, flow mechanics and wall shear stress in the great arteries of wildtype prenatal mouse [27]	2014	EFIC	heart	mouse	Segmentation	Level sets	3	Vascular modelling toolkit (VMTK) centerline prediction and geometry calculations	python (VMTK)	N/A
High-Resolution Episcopic Microscopy (HREM): A Tool for Visualizing Skin Biopsies [9]	2014	Eosin	skin biopsy	human	Object detection	Manual	N/A		Amira	N/A
High-resolution histological 3D-imaging: episcopic fluorescence image capture is widely applied for experimental animals [51]	2014	EFIC	various	Fish, mouse, chick, xenopus	Visualisation	N/A	N/A		Unknown	N/A
Multimodal Optical Microscopy Methods Reveal Polyp Tissue Morphology and Structure in Caribbean Reef Building Corals [52]	2014	EFIC	polyp	coral	Visualisation	Other	N/A		Imaris	N/A
Serial sectioning, multispectral imaging system for versatile biomedical applications [8]	2014	Cryo	organism	Fish	Visualisation	N/A	N/A		Unknown	N/A
Simultaneous dermal matrix, autologous split-thickness skin graft transplantation in a porcine wound model: A three-dimensional histological analysis of revascularization [53]	2014	Eosin	skin	pig	Segmentation	Manual	N/A		Amira	N/A
Tbx1 Coordinates Addition of Posterior Second Heart Field Progenitor Cells to the Arterial and Venous Poles of the Heart [54]	2014	Eosin	heart	mouse	Object detection	Manual	1	Phenotype counting	Osirix	N/A
The Development of Septation in the Four-Chambered Heart [55]	2014	Eosin	heart	mouse	Object detection	Manual	N/A		Unknown	N/A
Three-dimensional reconstruction of rat knee joint using episcopic fluorescence image capture [56]	2014	EFIC	knee joint	rat	Segmentation	Manual	5	Volume measurements	Amira, Osirix	N/A
A unique set of centrosome proteins requires pericentrin for spindle-pole localization and spindle orientation [57]	2015	EFIC	embryo	mouse	Visualisation	N/A	N/A		Unknown	N/A

Clarifying the morphology of the ostium primum defect [58]	2015	Eosin	ostium primium (Heart)	mouse	Object detection	Manual	N/A		Unknown	N/A
High-resolution episcopic microscopy (HREM): A useful technique for research in wound care [59]	2015	Eosin	Skin/dermal grafts	pig	Segmentation	Manual	N/A		Amira	N/A
iASPP, a previously unidentified regulator of desmosomes, prevents arrhythmogenic right ventricular cardiomyopathy (ARVC)-induced sudden death [60]	2015	Eosin	Heart	mouse	Visualisation	N/A	N/A		Unknown	N/A
Novel Jbts17 mutant mouse model of Joubert syndrome with cilia transition zone defects, cerebellar and other ciliopathy related anomalies [61]	2015	EFIC	brain, skeleton	mouse	Visualisation	Manual	2	Phenotype counting	ImageJ or Osirix	N/A
Physical factors affecting outflow facility measurements in mice [62]	2015	EFIC	eye	mouse	Segmentation	Manual	N/A		Amira	N/A
Postmortem high-resolution episcopic microscopy (HREM) of small human fetal hearts [63]	2015	Eosin	heart	human	Object detection	Manual	N/A		Unknown	N/A
Sequential binding of MEIS1, NKX2-5 on the Popdc2 gene: a mechanism for spatiotemporal regulation of enhancers during cardiogenesis [14]	2015	Eosin	heart	mouse	Visualisation	N/A	N/A		Unknown	N/A
Spatial change of cruciate ligaments in rat embryo knee joint by three-dimensional reconstruction [64]	2015	EFIC	cruciate ligament	rat	Segmentation	Manual	5	Length measurements	Amira	N/A
The CXCL12/CXCR4 Axis Plays a Critical Role in Coronary Artery Development [65]	2015	Eosin	Heart	mouse	Object detection	Manual	1	Phenotype counting	Imaris	N/A
The embryogenesis of the equine femorotibial joint: The equine interzone [66]	2015	Eosin	femorotibial interzone	horse	Visualisation	Manual	N/A		Unknown	N/A
3D modelling, tissue-level morphology of trapeziometacarpal joint [67]	2016	Unknown	TCM ligament	human	Segmentation	Manual	N/A		Unknown	N/A
ASPP2 deficiency causes features of 1q41q42 microdeletion syndrome [68]	2016	Eosin	brain	mouse	Object detection	Manual	1	Phenotype counting		N/A
Clarification of mammalian cloacal morphogenesis using high-resolution episcopic microscopy [69]	2016	EFIC	cloaca	mouse	Segmentation, Detection	Manual	5	Somite count	Amira	N/A

Comparing homologous microscopic sections from multiple embryos using HREM [70]	2016	Eosin	embryo	mouse	Object detection	Other	3	Affine deformation used to register multiple HREM datasets	ANTS	https://data.mouseimaging.ca/HREM/
Developmental considerations with regard to so-called absence of the leaflets of the arterial valves [71]	2016	Eosin	Arterial valves	human, mouse	Object detection	Manual	N/A		Unknown	DMDD
Functional joint regeneration is achieved using reintegration mechanism in *Xenopus laevis* [72]	2016	EFIC	elbow joint	xenopus laevis	Segmentation	Manual	N/A		Photoshop, Volocity	N/A
Genetic dissection of Down syndrome-associated congenital heart defects using a new mouse mapping panel [73]	2016	Eosin	heart	mouse	Segmentation	Manual	5	Volume measurements, shape factor analysis	ITKsnap, Volocity	N/A
High-throughput discovery of novel developmental phenotypes [74]	2016	Eosin	embryo	mouse	Object detection	Manual	N/A		Unknown	http://www.mousephenotype.org
Morphogenesis of myocardial trabeculae in the mouse embryo [75]	2016	Eosin	heart	mouse	Segmentation	Fractal	6	Fractal analysis	Osirix, Matlab	N/A
Selective subepicardial localization of monocyte subsets in response to progressive coronary artery constriction [7]	2016	Cryo	heart	rabbit	Segmentation	Intensity thresholding	5	Volume measurements	fiji	N/A
The embryological basis of subclinical hypertrophic cardiomyopathy [20]	2016	Eosin	heart	human, mouse	Segmentation	Fractal analysis	6	Fractal analysis, radial spoke thickness of wall	Osirix	N/A
A predictive model of asymmetric morphogenesis from 3D reconstructions of mouse heart looping dynamics [24]	2017	Eosin	heart	mouse	Segmentation	Manual	5, 7	Geometry measurements on resectioned imaged	Imaris, Icy, Blender	N/A
A staging system for correct phenotype interpretation of mouse embryos harvested on embryonic day 14 (E14. 5) [76]	2017	Eosin	Forelimb	mouse	Object detection	Manual	4	Phenotyping according to protocol in [9]	Amira, Osirix	DMDD
Cellular and morphological characterization of blastoderms from freshly laid broiler eggs [77]	2017	Eosin	embryo	chick	Object detection	N/A	N/A		Amira, ImageJ	N/A
Highly variable penetrance of abnormal phenotypes in embryonic lethal knockout mice [78]	2017	Eosin	embryo	mouse	Object detection	Manual	4	Phenotyping according to DMDD method	Amira, Osirix, Pixmeo, photoshop	DMDD

Morphology, topology and dimensions of the heart and arteries of genetically normal, mutant mouse embryos at stages S21–S23 [15]	2017	Eosin	heart	mouse	Segmentation, Detection	Manual	1, 5, 7	Phenotyping according to DMDD method, pipeline from [9]	Amira, Osirix	DMDD
Temporally Distinct Six2-Positive Second Heart Field Progenitors Regulate Mammalian Heart Development, Disease [79]	2017	Eosin	heart	mouse	Segmentation	Manual	N/A		Amira	N/A
Variation in the Three-Dimensional Histomorphometry of the Normal Human Optic Nerve Head With Age and Race: Lamina Cribrosa, Peripapillary Scleral Thickness, Position [80]	2017	EFIC	eye	human	Segmentation	Manual	5	Radis and thickness measurments	Visualizat ion Toolkit	N/A
WDR11-mediated Hedgehog signalling defects underlie a new ciliopathy related to Kallmann syndrome [81]	2017	Eosin	embryo	mouse	Object detection	Manual	1	Phenotype counting	Osirix	N/A
A simple setup for episcopic microtomy, a digital image processing workflow to acquire high-quality volume data, 3D surface models of small vertebrates [82]	2018	Other	organism	alytes obstetri cans; amphib ian Anura	Segmentation, Detection	Manual	7	Measurement of distances between fiducial markers after slice alignment	Amira, ImageJ	N/A
Comparative study of regenerative effects of mesenchymal stem cells derived from placental amnion, chorion and umbilical cord on dermal wounds [83]	2018	Eosin	skin wound	mouse	Segmentation	Manual	N/A		Amira	N/A
Effects of storage conditions on hatchability, embryonic survival and cytoarchitectural properties in broiler from young and old flocks [84]	2018	Eosin	egg	chick	Visualisation	N/A	N/A		Unknown	N/A
Identification of trophectoderm-derived Cripto as an essential mediator of embryo implantation [85]	2018	Eosin	embryo	mouse	Visualisation	Other	N/A		ImageJ, Amira	N/A
Placentation defects are highly prevalent in embryonic lethal mouse mutants [86]	2018	Eosin	embryo	mouse	Object detection	Manual	1	Phenotyping according to DMDD method	Amira, Osirix	DMDD
Quantification of the detailed cardiac left ventricular trabecular morphogenesis in the mouse embryo [87]	2018	Eosin	heart	mouse	Segmentation	Intensity thresholding, fractal analysis	5, 6	Measurements of volume and surface area, fractal analysis	ITKsnap, Seg3D	N/A

The dermal arteries in the cutaneous angiosome of the descending genicular artery [11]	2018	Eosin	Dermal arteries	human	Segmentation	Manual	5	Area and diameter measurments	Amira	N/A
The Tomato *BLADE ON PETIOLE* and *TERMINATING FLOWER* Regulate Leaf Axil Patterning Along the Proximal-Distal Axes [88]	2018	Eosin	leaf	Tomato plant	Visualisation	Manual	N/A		Fiji, Amira	N/A
A proof of concept study demonstrating that environmental levels of carbamazepine impair early stages of chick embryonic development [89]	2019	Eosin	embryo	chick	Visualisation	N/A	N/A		Amira, ImageJ	N/A
A Specific CNOT1 Mutation Results in a Novel Syndrome of Pancreatic Agenesis and Holoprosencephaly through Impaired Pancreatic, Neurological Development [90]	2019	Eosin	pancreas, forebrain	mouse	Segmentation	Manual	5	Volume measurements, phenotype count	Amira	DMDD
Diminution of pharyngeal segmentation and the evolution of the amniotes [91]	2019	Eosin	pharyngeal arches (brain)	human	Visualisation	Manual	N/A		Unknown	DMDD
Elastic instability during branchial ectoderm development causes folding of the Chlamydosaurus erectile frill [92]	2019	Eosin	erectile ruff	frilled dragon (Chlamydosaurus kingii)	Segmentation	Other	5	2D clustering	python, Amira	N/A
Furin, a transcriptional target of NKX2-5, has an essential role in heart development and function [93]	2019	Eosin	heart	mouse	Object detection	N/A	1	Defect counting	Osirix, Pixmeo	N/A
Haemodynamic stress-induced breaches of the arterial intima trigger inflammation, drive atherogenesis [94]	2019	Eosin	coronary artery	human	Segmentation	Intensity based	5	Distribution of iron plaques	Imaris	N/A
High-resolution episcopic microscopy enables three-dimensional visualization of plant morphology, development [95]	2019	Eosin	oil secretory cavity (citrus), adventitious root (persimmon)	plant woody plants: citrus fruit, persimmon rootstock	Visualisation	Manual	N/A		ImageJ, Amira	N/A

Myoarchitectural disarray of hypertrophic cardiomyopathy begins pre-birth [23]	2019	Eosin	heart orientiati on	mouse	Segmentation	Manual, Tensor	7	structure tensor method used to find orientation of caridomyocytes, wall thickness measured manually	Matlab, Fiji,	N/A
Pax9 is required for cardiovascular development and interacts with Tbx1 in the pharyngeal endoderm to control 4th pharyngeal arch artery morphogenesis [96]	2019	Eosin	pharynge al arches (heart)	mouse	Segmentation	Manual	1	Phenotype counting	Amira, Osirix	N/A
Standardised imaging pipeline for phenotyping mouse laterality defects and associated heart malformations, at multiple scales and multiple stages [97]	2019	Eosin	heart	mouse	Segmentation	Manual	1	Defect counting	Imaris, Icy, Blender	N/A
The Col4a2em1 (IMPC) Wtsi mouse line–lessons from the deciphering the mechanisms of developmental disorders (DMDD) program [98]	2019	Eosin	embryo	mouse	Segmentation, Detection	Manual	1	Phenotyping according to DMDD method	Amira, Osirix	DMDD
Asymmetric Point Spread Function Estimation and Deconvolution for Serial-Sectioning Block-Face Imaging [30]	2020	Fluoresce nce	brain + tumour	mouse	Segmentation	Tracing algorithm	5	PSF estimation, deconvolution, vessel radii measurements	Vaa3D, Amira, imageJ	N/A
Control of skeletal morphogenesis by the Hippo-YAP/TAZ pathway [99]	2020	Eosin	cleft palate	mouse	Segmentation, Detection	Intensity thresholding	5	Length measurements	Analyze Direct	N/A
Diverse species-specific phenotypic consequences of loss of function sorting nexin 14 mutations [100]	2020	Eosin	Embryo	mouse	Visualisation	N/A	N/A		Amira	N/A
Early Embryonic Expression of *AP-2α* Is Critical for Cardiovascular Development [101]	2020	Eosin	pharynge al arches (brain)	mouse	Segmentation, Detection	Manual	1	Phenotype counting	Amira	DMDD
High-resolution episcopic imaging for visualization of dermal arteries and nerves of the auricular cymba conchae in humans [102]	2020	Eosin	auricular cymba conchae	human	Segmentation	Manual	5	Volume and distance measurements	3D slicer	N/A
Identification, Morphogenesis of Vestibular Atrial Septal Defects [103]	2020	Eosin	heart	mouse	Object detection	Manual	N/A			DMDD
Loss of extreme long-range enhancers in human neural crest drives a craniofacial disorder [104]	2020	Eosin + X-gal	manible	mouse	Visualisation	N/A	N/A		Amira	N/A

Pax9 and *Gbx2* Interact in the Pharyngeal Endoderm to Control Cardiovascular Development [105]	*2020*	Eosin	pharyngeal arches (heart)	mouse	Segmentation	Manual	1	Phenotype counting	Amira, Osirix	N/A
Transient Nodal signaling in left precursors coordinates opposed asymmetries shaping the heart loop [106]	2020	Eosin + X-gal	Heart	mouse	Segmentation	Manual	5	Principle component analysis of shape parameters	Imaris, Icy, Matlab	N/A
Vegetative propagation of elite Eucalyptus clones as food source for honeybees (Apis mellifera); adventitious roots versus callus formation [107]	2020	Eosin	roots	eucalyptus tree	Visualisation	N/A	N/A		Amira	N/A
4D formation of human embryonic forelimb musculature [108]	2021	Eosin	upper forelimb	human	Object detection	N/A	N/A		ImageJ, Osirix	N/A
Artefacts in Volume Data Generated with High Resolution Episcopic Microscopy (HREM) [109]	2021	Eosin + acridine orange	embryo	mouse	Object detection	Manual	2	Artefact frequency count	Fiji	N/A
Cross-Modality Imaging of Murine Tumor Vasculature—a Feasibility Study [110]	2021	Eosin	tumour	mouse	Segmentation	Manual	4	Length and volume measurements	Amira	N/A
External surface anatomy of the postfolding human embryo: Computer-aided, three-dimensional reconstruction of printable digital specimens [111]	2021	EFIC	embryo	human	Segmentation	Active contour, Manual	N/A		ITK-SNAP	DMDD
Functional analysis of a gene-edited mouse model to gain insights into the disease mechanisms of a titin missense variant [112]	2021	Eosin	heart	mouse	Segmentation	Fractal analysis, manual	1, 4, 6	Length and volume measurements, fractal analysis	Osirix, Horos, Amira	DMDD
High Resolution Episcopic Microscopy for Qualitative and Quantitative Data in Phenotyping Altered Embryos and Adult Mice Using the New "Histo3D" System [113]	2021	Eosin + acridine orange	embryo	mouse	Segmentation, Detection	Manual	5	Volume measurements	Avizo	N/A
Histologic validation of optical coherence tomography-based three-dimensional morphometric measurements of the human optic nerve head: Methodology [114]	2021	EFIC	eye	human	Segmentation	Other	5	Surface fitting using manual landmarks	Visualization Toolkit	N/A
Hypoglossal Nerve Abnormalities as Biomarkers for Central Nervous System Defects in Mouse Lines Producing Embryonically Lethal Offspring [115]	2021	Eosin	embryo (Hypoglossal Nerve)	mouse	Segmentation, Detection	Manual	1	Phenotyping according to DMDD method	Amira and Osirix	DMDD

Imprinted Gene Expression, Function of the Dopa Decarboxylase Gene in the Developing Heart [116]	2021	EFIC	heart	mouse	Visualisation	N/A	N/A		Volocity	N/A
Msx1 haploinsufficiency modifies the Pax9-deficient cardiovascular phenotype [117]	2021	Eosin	embryo heart	mouse	Segmentation, Detection	Manual	N/A			yes
Multi-fluorescence high-resolution episcopic microscopy (MF-HREM) for three dimensional imaging of adult murine organs [118]	2021	Fluorescence	kidney, brain, tumour	mouse	Segmentation, Detection	Intensity based, Tracing algorithm	2, 5, 7	Deconvolution, vessel tracing, calculation of white matter orientation	Vaa3D, Amira, ImageJ, python, Matlab	IDR
Pathogenesis of Anorectal Malformations in Retinoic Acid Receptor Knockout Mice Studied by HREM [119]	2021	Eosin + acridine orange	embryo	mouse	Segmentation, Detection	Manual	N/A		Fiji	N/A
Reaction-diffusion in a growing 3D domain of skin scales generates a discrete cellular automaton [120]	2021	Eosin + acridine orange	skin	ocellated lizard	Segmentation	Intensity based	7	Geometric data from images used in reaction-diffusion modelling	Unknown	N/A
SMCHD1 has separable roles in chromatin architecture, gene silencing that could be targeted in disease [121]	2021	Eosin	embryo	mouse	Visualisation	Manual	4	Length measurements	OsirixMD	DMDD
Smooth Muscle Specific Ablation of CXCL12 in Mice Downregulates CXCR7 Associated with Defective Coronary Arteries, Cardiac Hypertrophy [122]	2021	Eosin	embryo	mouse	Segmentation	Manual	N/A		Amira	N/A
Miniseries 1—Part II: the comparative anatomy of the atrioventricular conduction axis [123]	2022	Eosin	heart	mouse	Visualisation	N/A	N/A			[124]
Analysis of placental arteriovenous formation reveals new insights into embryos with congenital heart defects [125]	2022	Eosin	placenta, heart	mouse	Segmentation	Manual	1, 4	Volume estimations using stereology method from Coan 2014	Amira, Horos	N/A
A three-dimensional, discrete-continuum model of blood flow in microvascular networks [16]	2022	Fluorescence	brain	mouse	Segmentation	Manual	4, 7	Skeletonised vessel network used for modelling blood flow	Amira	N/A
Bcar1/p130Cas is essential for ventricular development and neural crest cell remodelling of the cardiac outflow tract [126]	2022	Eosin	embryo heart	mouse	Segmentation	Manual	N/A	manual	Amira	N/A

Detailed characterizations of cranial nerve anatomy in E14.5 mouse embryos/fetuses and their use as reference for diagnosing subtle, but potentially lethal malformations in mutants. [127]	2022	Eosin	embryo cranial nerve	mouse	Segmentation	Manual	1, 4	Phenotyping according to DMDD method, nerve diameter measurements	Amira	DMDD
Detailed quantification of cardiac ventricular myocardial architecture in the embryonic, fetal mouse heart by application of structure tensor analysis to high resolution episcopic microscopic data [22]	2022	Eosin	embryo heart	mouse	Segmentation	Intensity based	6, 7	Structure tensor method used to find orientation of caridomyocytes	Fiji	N/A
Hinge point emergence in mammalian spinal neurulation [128]	2022	Eosin	embryo	mouse	Visualisation	N/A	N/A		Imaris 9.5.1	N/A
HREM, RNAseq, Cell Cycle Analyses Reveal the Role of the G2/M-Regulatory Protein, WEE1, on the Survivability of Chicken Embryos during Diapause [129]	2022	Eosin + acridine orange	embryo	chicken	Visualisation	N/A	N/A		Amira	N/A
Inhibition of TGFB pathway prevents short body size, cardiac defects in Nipbl-deficient mice, a mouse model of Cornelia de Lange syndrome [130]	2022	Eosin	heart	mouse	Visualisation	N/A	N/A		Imaris	N/A
Lem2 is essential for cardiac development by maintaining nuclear integrity [131]	2022	Eosin	heart	mouse	Visualisation	N/A	N/A			N/A
Miniseries 1—Part I: the Development of the atrioventricular conduction axis [132]	2022	Eosin	heart	human	Visualisation	N/A	N/A			[124]
Morphogenesis of the Mammalian Aortic Arch Arteries [133]	2022	Eosin	embryo	human, mouse	Segmentation, Detection	Manual	N/A		Amira	N/A
Novel Insights into the Etiology, Genetics, Embryology of Hypoplastic Left Heart Syndrome [134]	2022	EFIC	heart	mouse	Visualisation	N/A	N/A			N/A
PKN2 deficiency leads both to prenatal 'congenital' cardiomyopathy, defective angiotensin II stress responses [135]	2022	Eosin + acridine orange	embryo, heart	mouse	Visualisation	N/A	N/A		OsirisX	DMDD
The Nanog epigenetic remodeling complex was essential to vertebrate mesoderm evolution [136]	2022	Eosin	embryo	Axolotl	Visualisation	N/A	N/A			N/A
The venous system of E14.5 mouse embryos—reference data, examples for diagnosing malformations in embryos with gene deletions [137]	2022	Eosin + acridine orange	embryo	mouse	Segmentation, Detection	Manual	1, 5	Phenotype counting, length and diameter measurements	OsirisX	DMDD
